# A protocol for a rapid realist policy review (RRPR) of the impact of social determinants on self-harm and suicidal thoughts and behaviours in England

**DOI:** 10.1136/bmjopen-2025-110787

**Published:** 2025-12-24

**Authors:** Adam Hun Gen Chen, Maria Michail, Isabel Morales-Muñoz, Nicola Wright, Sarah-Jane Hannah Fenton

**Affiliations:** 1 School of Psychology, Institute for Mental Health, University of Birmingham, Birmingham, UK; 2 Centre for Youth Mental Health, The University of Melbourne, Melbourne, Victoria, Australia; 3 School of Health Sciences, University of Nottingham, Nottingham, UK; 4 Health Services Management Centre, University of Birmingham, Birmingham, UK

**Keywords:** Suicide & self-harm, Public health, MENTAL HEALTH

## Abstract

**ABSTRACT:**

**Introduction:**

Self-harm and suicidal thoughts and behaviours are a significant public health concern. While individual risk factors have been widely studied, the role of social determinants in shaping these outcomes remains underexplored within policy contexts. This Rapid Realist Policy Review aims to investigate how macro level (national) policies in England address the impact of social determinants of self-harm and suicidal thoughts and behaviours.

**Methods and analysis:**

This Rapid Realist Policy Review adapts the rapid realist review method to place policy documents at the centre of analysis. It will identify and extract relevant English policy documents (2002–2023) related to suicide, self-harm and mental health, using government and archival databases.

Policy documents will be mapped and categorised based on their pertinence to proximal and distal outcomes and social determinants. A predefined template will be used to extract and appraise data based on relevance, richness and rigour. Context-mechanism-outcome configurations will be developed, validated by content experts and synthesised into an initial programme theory. The review will follow Realist And Meta-narrative Evidence Syntheses: Evolving Standards for realist syntheses.

**Ethics and dissemination:**

This review does not require ethical approval due to the use of secondary sources. Findings will be disseminated via an open-access, peer-reviewed journal article. A summary of key recommendations will be produced with the expert stakeholder group to inform policy and practice.

**PROSPERO registration number:**

CRD420251057759.

**STRENGTHS AND LIMITATIONS OF THIS STUDY**

- This is the first study to use realist reasoning to explore the causal mechanisms of policies that address the impact of social determinants on self-harm and suicidal thoughts and behaviours.
- The use of the Pirkis *et al* model provides a comprehensive framework for categorisation and interpretation of social determinants within policy contexts.
- This study includes stakeholder consultation with subject experts and those with lived experience to ensure relevance and impact.
- Context-mechanism-outcome configurations and refined programme theories may be constrained due to the availability, quality and depth of policy documents.

## INTRODUCTION

### Background

 Suicide is one of the leading causes of death worldwide.^1^ One of the strongest predictors of suicide is self-harm,^2^ which refers to intentional self-poisoning or injury, irrespective of the apparent purpose.^3^ Approximately half of those who engage in self-harm will attempt suicide.^2 4^ In England, the rate of suicide (standardised by age) was 11.2 deaths per 100 000 in 2023, which showed an increase on previous years.^5^

In recent years, greater attention has been given to the social determinants of suicide (SDS) to address the complex and multifaceted nature of suicide and self-harm.^6^ SDS are defined as the ‘structures, processes and principles that influence societal decision making’ that have a direct, or indirect, impact on individual-level risk factors.^6^ They include the wider political, social, economic and environmental factors that are often influenced by societal systems and structures. A greater focus on these SDS may help identify relevant policy areas and encourage upstream measures for suicide prevention at a population level by tackling social inequalities.^7 8^

Recent umbrella reviews have identified a wide range of social determinants that increase the risk of suicide mortality, ideation or attempts. These included income and social protection, unemployment, environment and social inclusion, firearm accessibility, homelessness, experience of foster care, and experiences of childhood abuse and sexual assault.^9 10^ Findings regarding self-harm are more limited and inconsistent; however, child sexual abuse and working life conditions show a significant association with later self-harm.^11^ Further research is needed to clarify the relationship between other social determinants and self-harm. Overall, these findings suggest that social determinants play an important role in shaping risk for both suicide and self-harm.

Although evidence indicates that social determinants can influence the risk of both self-harm and suicide, several limitations remain. There is considerable methodological heterogeneity across the studies included in the umbrella reviews, limiting the robustness of findings. More significantly, none of the reviews examine suicide and self-harm together, making it difficult to build a comprehensive understanding of how social determinants influence self-harm and suicide.

In addition, little attention has been given to the role of policy, with much of the literature overlooking how policy addresses these determinants in which contexts and for whom. The lack of context-sensitive, theory-driven evidence in this area limits the ability of policymakers to establish the most impactful and cost-effective upstream measures to tackle self-harm and suicide risks across populations. This highlights a pressing need to understand how national-level policies address the impacts of social determinants on self-harm and suicidal thoughts and behaviours (STB) across the whole population.

### What this study adds

This research proposes an adaptation to the classic rapid realist review (RRR)^12^ with a central focus on policy documents, as well as linked or related evidence, in order to understand how national-level policies, both proximal and distal, address the impact of social determinants on self-harm and STB in England. In this review, proximal policies are those which explicitly address self-harm, STB and suicide mortality (eg, National Suicide Prevention Strategy in England), whereas distal policies do not directly address these outcomes, but are included within the Pirkis *et al*^6^ framework as a social determinant (eg, unemployment policies or housing policies).

Although widely used in health and social science research, systematic reviews are not well-suited to the proposed research. While they can identify and assess if programmes or interventions work, they are limited in understanding why, how or for whom an intervention (or in this case, policy) works.^13^ The realist approach enables an exploration of the contextual factors and generative mechanisms, which refer to the underlying processes or responses to policies that lead to the outcome (self-harm, STB or suicide mortality) within a specific context being addressed. In exploring the generative mechanisms and contextual factors, it examines how policies in England address SDS and self-harm; thus, contributing towards the literature on the policy dimensions of SDS and ensuring self-harm is also included as a central outcome.

The realist method also facilitates an appraisal of actions, not solely based on evidence, but through answering “how and for whom, in what circumstances and why” do policies work using context-mechanism-outcome (CMO) statements (eg, “In this context, this mechanism generates this outcome”).^14^ Moreover, it allows for the discussion and deeper assessment of quality through an examination of relevance, richness and rigour, which is more appropriate to the review material. Reviewing data based on contribution towards theory building/testing, contextual/conceptual detail and credibility and trustworthiness.^15^

### A policy-focused approach

The review adopts the Pirkis *et al*^6^ public health model of suicide, which provides a conceptual lens to understanding and contextualising the role of SDS. The model distinguishes between proximal individual-level risk factors, such as sociodemographic and contextual influences, and broader, upstream structural factors, that is, political, social and economic structures. While it acknowledges the relevance of individual vulnerabilities, the distinction of social determinants allows for a more policy-oriented focus on the broader structural conditions that influence self-harm and STB.

The model will inform multiple components of this review, including the definition and categorisation of SDS. It offers an initial framework for the review, providing conceptual clarity by helping to identify and categorise policies for further synthesis. Using the model, SDS include macroeconomic policies (eg, austerity or regressive taxation); public policies (eg, those related to alcohol regulation); social policies (eg, educational or employment policies); and legislative or regulatory frameworks (eg, the Housing Act, 2004). Additional areas of focus encompass determinants such as the local environment, cultural and societal values, social cohesion and social capital, and commercial determinants. Together, this framework enables a focused, structured and policy-relevant analysis of how national strategies have addressed the social determinants of self-harm and STB. This further contributes to knowledge creation, as policies are often reviewed individually or in siloed approaches relating to the system they inform, that is, health or education. This research looks at a specific issue across macro (national) policies to inform understanding.

### Aim

This Rapid Realist Policy Review (RRPR) aims to explore how national-level policies in England address the impact of social determinants on self-harm and STB across the whole population.

The specific research questions are:

- What proximal and distal policies exist to address the impact of social determinants on self-harm and STB?
- What priorities/actions are identified within policy to address the social determinants that increase the risk of self-harm and STB?
- What evidence underpins/supports policies/actions aimed at addressing the social determinants of self-harm and STB?
- What is the focus of policy/actions (ie, for whom, in what circumstances and why is the policy intended to work?)

This review explores how national policies address the impact of social determinants across the wider population. It will also highlight if, and how, these policies distinctly address the needs of young people (16 to 24 years of age, as defined by National Health Service England^16^). Young people are a priority group because suicide is one of the leading causes of death in this age range,^1^ and exposure to social determinants could have adverse influences.^9 10^

## METHODS

### Patient and public involvement

Stakeholder involvement is central to realist research.^12^ A key stakeholder group is young people, and to ensure their views are heard, we will leverage the expertise of the Institute for Mental Health Youth Advisory Group (YAG). Within this review, the YAG is consulted to provide feedback on the proposed research questions and identify priority areas for future investigation. The YAG consists of young people aged 18–25 who have lived or living experiences, or interest in mental health, who play an active role in shaping and challenging youth mental health research.^17^

This review draws on two forms of stakeholder expertise—subject experts and those with lived experience. The YAG provides the lived or living experience expertise. Their insights will sit alongside other subject expertise stakeholders, including content experts such as policy specialists and suicide prevention researchers, to sense-check programme theories (hypothetical statements). Additional details of stakeholder involvement are provided in Step 7.

### Rapid realist policy review

**Figure 1:**
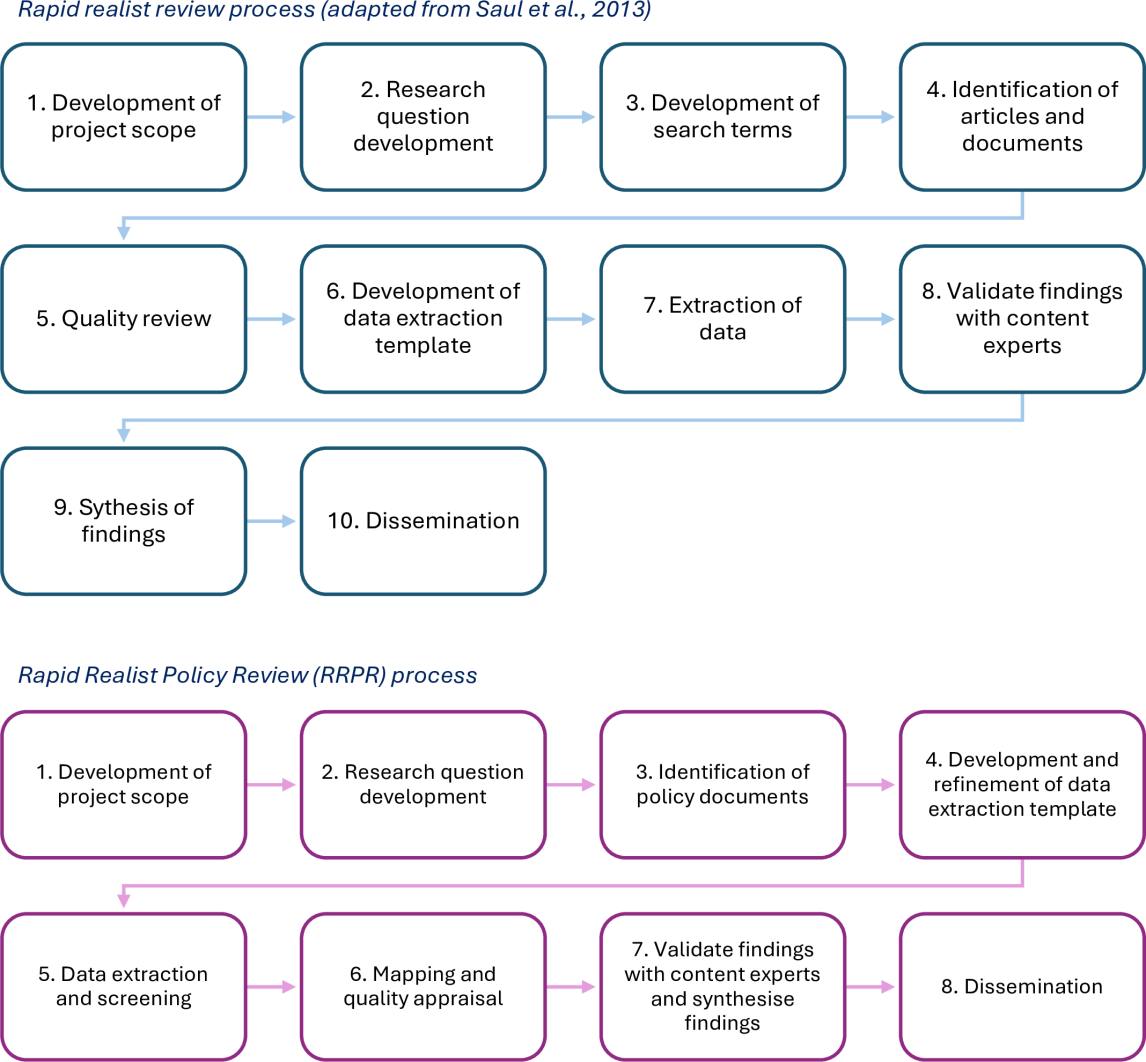
Comparison between the original RRR process by Saul *et al*^12^ and the adapted RRPR process. The top diagram illustrates Saul *et al*’s RRR approach; the bottom diagram presents the adapted RRPR process tailored for policies. RRPR, Rapid Realist Policy Review; RRR, rapid realist review.

This project will adapt Saul *et al*’s^12^ approach to RRR to develop a theory-driven method to search and analyse policy documents (see figure 1). The RRR approach is iterative, and there may be potential adaptations or changes throughout the process.^18^ Similar adaptations of the rapid realist methodology have been undertaken for policy analysis, such as the Accelerated Parallelised Realist-Informed Literature review (APRIL) method.^19^ The timeframe for the review, from conception to completion, is from February 2025 through to January 2026.

### Steps 1 and 2: Development of project scope and research question development

This study responds to existing gaps in the current literature, using a policy-focused approach guided by Pirkis *et al*’s^6^ model, to examine how policies in England address the impact of SDS on self-harm and suicidal thoughts and behaviours. A knowledge expert in policy and realist methodology, recruited through the partnership and networks of the supervisory team, supports the development of the scope and research questions, including the methodological adaptations. Consultations with the supervisory team and knowledge expert inform the feasibility of the review, the realist focus on ‘how and for whom, in what circumstances and why’ and the outcomes of interest (self-harm, STB and suicide mortality). These discussions shape the review aim of how policies address the impact of social determinants on self-harm and STB, and research questions. To address the complexities of identifying grey literature, an information specialist advises on the appropriate tools, databases and strategies for locating grey literature, such as the UK Government Website (https://www.gov.uk/search/policy-papers-and-consultations).

Three search terms will be used: ‘suicide’, ‘self-harm’ and ‘mental health’. The search term ‘mental health’ helps to delineate mental health policy documents from broader general health policies and will improve the relevance of the search and reduce any potential noise through the acknowledgement of mental health as a distinct policy domain; it may also help capture broader policies that relate to mental health in non-health-related searches.

Additionally, to maintain a clear policy focus, searches will be restricted to a defined policy window. This defined period will span from the first National Suicide Prevention Strategy for England, published in 2002, to the most recent, published in 2023. In doing so, it narrows the review’s scope to a manageable but relevant set of policies. Thus, refining the breadth of analysis and enabling a targeted examination of how national policies between key suicide prevention strategies have addressed the broader political, social, economic and environmental factors that influence self-harm and STB.

The inclusion and exclusion criteria for policy documents are presented in table 1:

**Table 1:** Inclusion and exclusion criteria for the identification and screening of policy documents

Inclusion	Exclusion
Setting: England Age range: no specified criteria required Timeframe: Policy documents from September 2002 (aligning with the publication of the first National Suicide Prevention Strategy for England) to September 2023 (the latest publication). National ‘policy documents’: Strategy documents and plans; White papers; Statutory guidance	Policy documents outside England Policy documents not within 2002–2023 Regional/local policy documents in England Government responses Annual/progress reports Calls for evidence Fact sheets and figures Consultation documents Memorandums and communiqués

### Step 3: Identification of policy documents

A pilot search for ‘suicide’ will be conducted using the UK Government website to test the inclusion and exclusion criteria listed in table 1. Filters will be applied to document type (policy paper) and timeframe (September 2002 to September 2023), and no filter for ‘topic’, ‘organisation’ or ‘world location’. Filters are applied to refine the number of relevant documents and remove documents in line with the inclusion and exclusion criteria.

Following the pilot, three separate searches will be run using the established search terms using the UK Government website. Additional targeted searches will identify archived or earlier versions of documents not on the UK Government website, via the Parliamentary Library and the National Archives (https://www.nationalarchives.gov.uk/). If the prior methods do not identify documents, a targeted Google search will be undertaken.

Further documents will be located with the bibliographic database Social Policy & Practice, which will be accessed through Ovid with the same search terms, timeframes and filters for policy documents. This will be used in combination with the primary search to account for the ephemeral nature of grey literature, which lacks formal indexing and potential ‘link rot’.

A final search will be conducted through ‘snowball searches’ with the help of knowledge and content experts in policy and suicide prevention.^12^ These will be sought via The Health Foundation, Centre for Mental Health and Institute for Health Equity. The inclusion of these organisations is on the basis of relevance to mental health and other social determinants.

### Steps 4 and 5: Development and refinement of the data extraction template and data extraction and screening

The extraction template will be developed as a table in Microsoft Excel. Data extracted will include: the policy citation (the title, web address, publication date, department(s) involved), information on the population of focus within the policy (eg, whether it focuses on specific populations), and which social/commercial determinants and individual factors are prioritised (derived from the categories in the Pirkis *et al*^6^ framework). In addition, outcome-related data (ie, self-harm or suicidal thoughts and behaviour or mortality), recommendations/action plans for policies, and evidence used to support the policy, which is expected to include cited research, will be extracted. Further extraction details will be developed iteratively, in consultation with mental health policy experts and subject-specific experts who will also form the stakeholder group.

Policy documents will then be extracted and screened in accordance with the inclusion/exclusion criteria, which will also serve as an initial assessment of relevance. Relevant details of those who are screened will be extracted from the policy documents into the predefined template.

### Stage 6: Mapping and quality appraisal

Mapping and quality appraisal will work in tandem as an iterative process. Mapping will group and categorise data on SDS, from the policy documents. This will be compared with the SDS outlined by Pirkis *et al.*^6^ The aim of this is to identify a range of social determinants addressed in policies. The second step will then involve the identification and classification of outcomes as either proximal or distal to self-harm, STB and suicide mortality.

The extraction of data from policy documents, in conjunction with the structured template and categorisation by social determinants and proximal/distal outcomes, will help cluster data related to self-harm and STB. This process enables the identification of key components relevant to the context, mechanism and outcomes. We define context as the background conditions in which policy operates, including social rules, values and interrelationships, as well as the resources available that may support or constrain mechanisms.^20 21^ Mechanisms are understood as generative (they produce outcomes) underlying processes that can be distinguished into (material) resources^22 23^ such as increased training or policy initiatives, and reasoning, which result in changes in behaviour or thinking.^20^ Outcomes refer to the intended, unintended or unexpected results of mechanisms, such as a better understanding of suicide (outcome) because of improved training (mechanism).^21^

Appraisal of the quality of evidence will adapt Dada *et al*’s^15^ appraisal method to evaluate data based on its relevance, richness and rigour (ie, trustworthiness and reliability) in relation to the research questions.

The quality review process will be iterative, as sources may be revisited. For example, if the policy document is ranked as ‘low’, it may be excluded from the review but will be retained for consideration in relation to the development of initial programme theories to support, refine or refute theories.^15^

The screening process will determine the relevance of policy documents, that is, whether they meet the inclusion/exclusion criteria. These documents will be assessed to ascertain whether they address self-harm, suicidal thoughts and behaviour directly or indirectly through related social determinants (proximal/distal); this will assess their ‘richness’. The final assessment will be against rigour, which will include trustworthiness and reliability and will focus on ‘how, for whom, in what circumstances and why?’ and will be appraised through the utilisation of the following questions:

(how) Is there a discussion of the mechanisms (reasoning, processes or resources) triggered by the policy? (Y/N)
(whom) Are the groups or individuals addressed outlined? (Y/N)
(what circumstances) Is there a discussion of the enabling or constraining factors? (Y/N)
(why) Is there reasoning/rationale behind the policy discussed, ie, evidence backing or theory discussion? (Y/N)
Are the proposed outcomes/actionable points measurable? (Y/N)
How detailed are the proposed outcomes/actionable points?
  (5, clearly specifies how it will be implemented, who it targets, what circumstances influence them and why; 0, vague and non-specific)

Evidence will be ranked from 0 to 10, with 10 meaning a high level of rigour, and 0, meaning low rigour. A second reviewer will review 10% of documents to ensure consistency. Subsequent discussions with the supervisory team will moderate any disagreement.

### Step 7: Validate findings with content experts and synthesis of findings

CMO configurations will be developed for each theme. Causal statements will be developed from the discrete context; mechanisms and outcomes will be identified within policy documents and conveyed as ‘if … then’ statements. Once this has been completed, patterns, also known as demi-regularities, will be identified, and causal statements will be combined.^24^ These will then be grouped by theme (eg, economic strain, stigma and discrimination, housing instability, alcohol and substance use regulation) into CMO configurations. These will be used to develop and refine initial programme theories (IPT), which will be validated by the stakeholder group. This is to ensure the findings reflect the knowledge and experiences of policy experts and lived experiences and identify gaps.

To ensure that the IPTs are accurately grounded in real-world experiences, they will be reviewed by and refined in partnership with a stakeholder group comprising individuals with lived experience and knowledge experts. Through their input, the IPTs may be confirmed, refined or refuted consistent with the data validation and theory testing approach outlined by Saul *et al.*^12^

### Step 8: Dissemination

The review results will adopt the RAMESES (Realist And Meta-narrative Evidence Syntheses: Evolving Standards) publication standards.^25^ The protocol has been registered with the PROSPERO (CRD420251057759). Findings will be disseminated via an open-access, peer-reviewed journal article. A summary of key recommendations will be produced with the stakeholder group to inform policy and practice.

### OUTCOMES

The primary outcome of the RRPR is to develop an initial programme theory to understand why, how, for whom and in what circumstances national policies address the impacts of social determinants of self-harm and STB in England. The RRPR contributes to methodological advancement by refining and adapting the traditional RRR literature review methods to better suit the context of policy documentary analysis.

### ETHICS AND DISSEMINATION

This review does not require ethical approval as it draws exclusively on secondary sources available in the public domain. Dissemination plans are outlined in Step 8 of the RRPR methodology. The findings will be shared in accordance with reporting standards and published in an open-access, peer-reviewed journal.

## Data Availability

No data are available.

## References

[1] World Health Organization (2025). Suicide. https://www.who.int/news-room/fact-sheets/detail/suicide.

[2] Hawton K, Bergen H, Cooper J (2015). Suicide following self-harm: findings from the Multicentre Study of self-harm in England, 2000-2012. J Affect Disord.

[3] National Institute for Health and Care Excellence (2022). National Institute for Health and Care Excellence. Self-harm: assessment, management and preventing recurrence (NG225).

[4] Rodway C, Tham S-G, Ibrahim S (2016). Suicide in children and young people in England: a consecutive case series. Lancet Psychiatry.

[5] Office for National Statistics (2023). Suicides in England and Wales.

[6] Pirkis J, Dandona R, Silverman M (2024). Preventing suicide: a public health approach to a global problem. Lancet Public Health.

[7] Shand F, Yip D, Tye M (2020). The Impact of Social Determinants on Suicide and How Policy Settings Can Help.

[8] National Action Alliance for Suicide Prevention (2025). Moving Suicide Prevention Upstream.

[9] Na PJ, Shin J, Kwak HR (2025). Social Determinants of Health and Suicide-Related Outcomes: A Review of Meta-Analyses. JAMA Psychiatry.

[10] Gallagher K, Phillips G, Corcoran P (2025). The social determinants of suicide: an umbrella review. Epidemiol Rev.

[11] McClelland H (2023). Social determinants associated with non-accidental self-harm: a rapid umbrella review.

[12] Saul JE, Willis CD, Bitz J (2013). A time-responsive tool for informing policy making: rapid realist review. Implement Sci.

[13] Hunter R, Gorely T, Beattie M (2022). Realist review. Int Rev Sport Exerc Psychol.

[14] The RAMESES II Project (2017). What is a mechanism? what is a programme mechanism?.

[15] Dada S, Dalkin S, Gilmore B (2023). Applying and reporting relevance, richness and rigour in realist evidence appraisals: Advancing key concepts in realist reviews. Res Synth Methods.

[16] NHS England (2024). Age: NHS England.

[17] University of Birmingham Institute for mental health youth advisory group birmingham. https://www.birmingham.ac.uk/research/centres-institutes/mental-health/youth-advisory-group.

[18] Pawson R, Greenhalgh T, Harvey G (2005). Realist review - a new method of systematic review designed for complex policy interventions. *J Health Serv Res Policy*.

[19] Aunger J, Ungureanu B, Posaner R Accelerated Parallelised Realist-Informed Literature review (APRIL): a novel method for informing policy decision-making in accelerated (<1 month) timescales. International Journal of Qualitative Methods [Forthcoming].

[20] Pawson R, Tilley N (1997). Realistic Evaluation.

[21] De Weger E, Van Vooren NJE, Wong G (2020). What’s in a Realist Configuration? Deciding Which Causal Configurations to Use, How, and Why. Int J Qual Methods.

[22] Sayer RA (2010). Method in social science: a realist approach: routledge.

[23] Dalkin SM, Greenhalgh J, Jones D (2015). What’s in a mechanism? Development of a key concept in realist evaluation. Implement Sci.

[24] Jagosh J, Macaulay AC, Pluye P (2012). Uncovering the benefits of participatory research: implications of a realist review for health research and practice. Milbank Q.

[25] Wong G, Greenhalgh T, Westhorp G (2013). RAMESES publication standards: realist syntheses. J Adv Nurs.

