# Supplementary material for: A protocol for a rapid realist policy review (RRPR) of the impact of social determinants on self-harm and suicidal thoughts and behaviours in England
Source: BMJ Open. 2025 Dec 24;15(12):e110787. doi: 10.1136/bmjopen-2025-110787 (PMC12742089; doi:10.1136/bmjopen-2025-110787)
Supplement: Supplementary file 1 [file bmjopen-2025-110787.reviewer_comments.pdf]

PEER REVIEW HISTORY

BMJ Open publishes all reviews undertaken for accepted manuscripts. Reviewers are asked to complete a checklist review form and are provided with free text boxes to elaborate on their assessment. These free text comments are reproduced below.

ARTICLE DETAILS

Title (Provisional)

A Protocol for a Rapid Realist Policy Review (RRPR) of the Impact of Social Determinants on Self-Harm and Suicidal Thoughts and Behaviours in England

Authors

Chen, Adam Hun Gen; Michail, Maria; Morales-Muñoz, Isabel; Wright, Nicola; Fenton, Sarah-Jane Hannah

---

VERSION 1 - REVIEW

**Reviewer**	**1**
**Name**	**Das, Soumitra**
**Affiliation**	**National Institute of Mental Health and Neuro Sciences**
**Date**	**03-Nov-2025**
**COI**	**None**

This is a step towards doing the policy reviews. Each part of the protocol is clearly mentioned. Please dictate the need for involving peer group, what will be the process in details.

**Reviewer**	**2**
**Name**	**MacPhee, Maura**
**Affiliation**	**The University of British Columbia**
**Date**	**18-Nov-2025**
**COI**	**None**

Your protocol is a very interesting one. I have some minor requests before publication acceptance:

1. Although your inclusion criteria do not specify an age range, you're using a Youth Advisory Group. What about other age groups with lived experience?

2. Your search range is 2002-2023. Why did you select this search range?

3. On page 12, around line 28, I recommend providing better definitions, description and examples of Cs, Ms and Os. Not all readers may be familiar with realist terminology. I think your description of a Mechanism is especially vague.

VERSION 1 - AUTHOR RESPONSE

Reviewer 1

This is a step towards doing the policy reviews. Each part of the protocol is clearly mentioned. Please dictate the need for involving peer group, what will be the process in details.

Thank you for your comment on clarifying peer group involvement. As per the Rapid Realist Review (RRR) approach devised by Saul et al. (2013), stakeholder involvement is tantamount to the expedited nature as well as data validation. We have further clarified this in our manuscript in Step 7 (page 13): validate findings with context experts and synthesis of findings:

"To ensure that the IPTs are accurately grounded in real-world experiences, they will be reviewed by and refined in partnership with a stakeholder group comprising of individuals with lived experience and knowledge experts. Through their input the IPTs may be confirmed, refined, or refuted consistent with the data validation and theory testing approach outlined by Saul et al.[12]"

Reviewer 2

Although your inclusion criteria do not specify an age range, you're using a Youth Advisory Group. What about other age groups with lived experience?

The Youth Advisory Group (YAG) forms one aspect of our stakeholder group, alongside context experts who will also validate the programme theories. This does not diminish the value of other age groups with lived experience. We listed the YAG as a named stakeholder group, as their involvement in this policy review is important given the increase rates of suicide among those aged 16 to 25. In addition to the changes above, we have clarified our reasonings (page 7):

"*Stakeholder involvement is central to realist research.[12] A key stakeholder group is young people and to ensure their views are heard we will leverage on the expertise of the Institute for Mental Health Youth Advisory Group (YAG). Within this review, the YAG are consulted to provide feedback on the proposed research questions and identify priority areas for future investigation. The YAG consists of young people aged 18-25 who have lived or living experiences, or interest in mental health, who play an active role in shaping and challenging youth mental health research.[17]*

This review draws on two forms of stakeholder expertise – subject experts and those with lived experience. The YAG provide the lived or living experience expertise. Their insights will sit alongside other subject expertise stakeholders, including content experts such as policy specialists and suicide prevention researchers, to sense-check programme theories (hypothetical statements). Additional details of stakeholder involvement are provided in Step 7." (page 13).

Your search range is 2002-2023. Why did you select this search range?

In response to the reviewer's comment, we have further clarified our reasoning (page 11) for this specific search range.

"Additionally, to maintain a clear policy focus, searches will be restricted to a defined policy window. This defined period will span from the first National Suicide Prevention Strategy for England, published in 2002, to the most recent, published in 2023. In doing so, it narrows the review's scope to a manageable but relevant set of policies. Thus, refining the breadth of analysis and enabling a targeted examination of how national policies between key suicide prevention strategies have addressed the broader political, social, economic and environmental factors that influence self-harm and STB."

On page 12, around line 28, I recommend providing better definitions, description and examples of Cs, Ms and Os. Not all readers may be familiar with realist terminology. I think your description of a Mechanism is especially vague.

Thank you for the comment. We recognise that realist terminology can be difficult to understand to those not familiar and thus have strengthened and clarified our definitions of Context, Mechanisms and Outcomes (page 11).

"The extraction of data from policy documents, in conjunction with the structured template and categorisation by social determinants and proximal/distal outcomes, will help cluster data related to self-harm and STB. This process enables the identification of key components relevant to the context, mechanism, and outcomes. We define Context as the background conditions in which policy operates, including social rules, values, and interrelationships, as well as the resources available that may support or constrain mechanisms.[20, 21] Mechanisms are understood as generative (they produce outcomes) underlying processes that can be distinguished into (material) resources [22 23] such as increased training or policy initiatives, and reasoning, which result in changes in behaviour or thinking.[20] Outcomes refer to the intended, unintended, or unexpected results of mechanisms, such as a better understanding of suicide (Outcome) because of improved training (Mechanism).[21]"

References

Saul, J. E., Willis, C. D., Bitz, J., & Best, A. (2013). A time-responsive tool for informing policy making: rapid realist review. *Implementation science : IS*, *8*, 103. https://doi.org/10.1186/1748-5908-8-103
